# TLR2 Elicits IL-17-Mediated RANKL Expression, IL-17, and OPG Production in Neutrophils from Arthritic Mice

**DOI:** 10.1155/2014/643406

**Published:** 2014-03-16

**Authors:** Viktoriya Milanova, Nina Ivanovska, Petya Dimitrova

**Affiliations:** Department of Immunology, Institute of Microbiology, Bulgarian Academy of Sciences, 26 Georgi Bonchev street, 1113 Sofia, Bulgaria

## Abstract

We investigated the ability of neutrophils to express receptor activator of nuclear factor kappa-B ligand (RANKL), to secrete osteoprotegerin (OPG), and to produce IL-17. Arthritis was induced by intra-articular injection of zymosan, a ligand for Toll-like receptor 2 (TLR2). Frequencies of neutrophils in bone marrow (BM), blood and synovial fluid (SF), receptor expression, and cytokine production were evaluated by flow cytometry. 1A8 antibody (1A8 Ab) was used to deplete neutrophils in zymosan-injected SCID mice. IL-17, RANKL, and OPG amounts in SF, serum, or cell cultures were determined by ELISA. The development of arthritis was associated with increased secretion of IL-17, RANKL, and OPG in serum and SF, elevated frequencies of Ly6G^+^CD11b^+^ cells in BM, blood, and SF and upregulated RANKL expression. Both IL-17 and OPG were absent in serum and SF after neutrophil depletion; therefore we assume that they were released by neutrophils. In vitro blood Ly6G^+^CD11b^+^ cells from arthritic mice produced spontaneously IL-17, IFN-*γ*, and OPG and expressed RANKL. This phenotype was sustained by IL-17. TLR2 engagement increased IL-17 and IFN-*γ* production, potentiated IL-17-mediated RANKL expression, and inhibited OPG secretion. We conclude that TLR2 regulates the destructive potential of neutrophils and its targeting might limit joint alterations in arthritis.

## 1. Introduction

Neutrophils are the most abundant cells in SF at the initial phase of rheumatoid arthritis (RA). They deliver signals or/and release factors regulating the functions of synovial fibroblasts, chondrocytes, osteoclasts, and other inflammatory cells like monocytes, T and B cells, dendritic cells, and NK cells. Neutrophils from RA patients are functionally different from those of healthy donors (reviewed by [1]). They have an active NF-*κ*B signaling pathway and produce considerable amounts of reactive oxygen species and tumor necrosis factor (TNF)-*α* [1]. Their cytoplasm is enriched with granules containing proteases, phospholipases, defensins, and myeloperoxidase (just before being reviewed in [2]). The release of all these factors in SF induces collagen and proteoglycan depletion, receptor shedding, cytokines degrading, and activation of cytokine precursors. Neutrophils from RA patients have also delayed apoptosis and are susceptible to stimulation via TLRs and receptors for complement fragments, growth factors, and cytokines (reviewed by [3]).

Among the members of the Toll-like receptors, family is TLR2. The receptor interacts with microbial lipopeptides such as peptidoglycan from gram-positive bacteria, lipoarabinomannan from mycobacteria, and zymosan (ZY) from yeast cell wall. TLR2 has an extracellular domain with leucine-rich repeats and a conservative intracellular Toll/IL-1 receptor (TIR) domain. TLR2 forms homodimers or heterodimers with TLR1 or TLR6 [4]. Its downstream pathways involve myeloid differentiation factor 88 (MyD88), c-Jun N-terminal kinase, NF-*κ*B, and phosphatidylinositol 3-kinase (PI3K), which promotes NF-*κ*B-dependent transcription too (reviewed by [5]). Various studies have shown the role of TLR2 signaling for the development of arthritis (reviewed by [6]). Among them are investigations on TLR2-deficient mice describing a direct suppression of neutrophils function in these animals and better outcome from arthritis [7, 8].

IL-17 is characteristic for the early stage of arthritis and plays a role in various inflammatory and autoimmune pathologies [9–11]. Elevated IL-17 mRNA expression in SF can predict the progression of joint damage and occurs before the disease onset [12, 13]. The overexpression of IL-17 promotes the development of collagen-induced arthritis, while IL-17 neutralization inhibits bone erosion and cartilage damage [14, 15]. IL-17 interferes with RANKL signaling pathway, osteoclastogenesis and maintains matrix turnover and cartilage destruction, especially in the presence of TNF-*α* [16, 17]. The cytokine promotes not only joint inflammation but also a bone-protective potential of neutrophils in periodontal disease [18].

Neutrophils from RA patients express RANKL and secrete a decoy RANKL receptor, OPG [19]. We have found abrogated RANKL expression on neutrophils that contributes to better outcome from collagen-antibody-induced arthritis in properdin-deficient mice [20]. In a model of collagenase-induced osteoarthritis glucosamine inhibits bone destruction and decreases the number of RANKL-bearing neutrophils in SF [21]. Our previous studies involving patients with osteoarthritis show altered TNF-*α* production in response to TLR2 stimulation and elevated TLR2 and RANKL expression on blood neutrophils [22, 23]. In the present work we investigate the bone-destructive activity of Ly6G^+^CD11b^+^ cells in TLR2 ligand driven arthritis. To confirm that neutrophils directly participate in bone resorption monoclonal 1A8 Ab recognizing that Ly6G was administrated to zymosan-injected SCID mice. Ly6G^+^CD11b^+^ cells were depleted in circulation and we measured the concentrations of IL-17, RANKL, and OPG in SF and serum. We examined IL-17 and IFN-*γ* production of blood neutrophils by flow cytometry and we evaluated the effect of IL-17 and TLR2 stimulation on cytokine production, RANKL expression, and OPG secretion in these cell cultures.

## 2. Materials and Methods

### 2.1. Animals

All experiments were approved by the Animal Care Committee at the Institute of Microbiology, Sofia, in accordance with the National and European Guidelines. BALB/c and SCID (CB17) mice were purchased from the Charles River Laboratories (USA), kept under standard conditions of a 12–12 hours light-dark cycle, and fed with a laboratory diet and water ad libitum. Mice (weigh 20–22 g) were anesthetized by intraperitoneal injection (i.p.) of sodium pentobarbital (50 mg/kg; Sigma-Aldrich, Munich, Germany) supplemented with buprenorphine hydrochloride analgetic (0.1 mg/kg; Sigma-Aldrich).

### 2.2. Arthritis

BALB/c mice were injected intra-articularly (i.a.) at ankles or knees with 10 *μ*L of zymosan suspension (20 mg/mL; Sigma-Aldrich) or 10 *μ*L of endotoxin-free phosphate-buffered saline (PBS; control group). To deplete neutrophils in SCID mice monoclonal 1A8, Ab (endotoxin free, 100 *μ*g in 200 *μ*L per mouse; Biolegend, London, UK) was administered i.p. at days −2, +2, and +4. At day 0 SCID mice were injected i.a. with PBS (PBS + Ab group) or zymosan (ZY + Ab group). The development of disease for 7 days was compared to untreated PBS- (PBS group) or zymosan- (ZY group) injected SCID mice. To monitor cell depletion Ly6G^+^CD11b^+^ cell, frequencies were assessed in BM at days −2, +2, and +4.

### 2.3. Histology

At day 7 of arthritis, ankle or knee joints were dissected, fixed in 10% paraformaldehyde/PBS, decalcified in 5% nitric acid for 1 week, dehydrated, embedded in paraffin, cut, and stained with hematoxylin and eosin (H&E) or Safranin O [24]. The degree of injury was graded by a three score system applied for cell infiltration and proteoglycan loss (score 0—no abnormality; score 3—severe abnormalities) and determined by two independent observers using light microscopy (Leica Microsystems, Wetzlar, Germany). Cartilage erosion was expressed as the percentage of impaired cartilage from the total cartilage surface and was determined after photo capturing by a DS-Ri1 Nikon camera (Nikon Instruments Europe, Amstelveen, The Netherlands) and image analyses by ImageJ 1.42 software (Research Services Branch, NIH, Bethesda, MD, USA).

### 2.4. ELISA Assay

SF was harvested from ankles or knees by lavage with 25 *μ*L of PBS containing 1 mM EDTA (Sigma-Aldrich). Serum was obtained after centrifugation of collected blood. RANKL, OPG, and IL-17 were quantified in SF, serum, or culture supernatants by ELISA kits from Abcam (Cambridge, UK; detection limit < 4 pg/mL and of < 1 pg/mL, resp.) and from Biolegend (London, UK; detection limit < 8 pg/mL). The samples were assayed in triplicate. The concentrations of RANKL, OPG, and IL-17 were calculated from a standard curve of the respective recombinant mouse protein using Gen5 Data Analysis Software (BioTek Instruments, Bad Friedrichshall, Germany).

### 2.5. Cell Isolation and Phenotype

Synovial cells were isolated by centrifugation of SFs. Peripheral cells were obtained from heparinized blood after Histopaque (Sigma-Aldrich) density gradient centrifugation. BM cells were collected from the tibia and femur. Exclusion dye staining with 0.05% Trypan blue showed more than 95% viable cells in isolated populations. After washing, cells were resuspended at 1 × 10^5^/mL in 2% FCS/PBS and incubated with Abs against mouse Ly6G (clone 1A8; Biolegend), CD11b (clone M1-70; Biolegend), and CD69 (clone H1.2F3; BD Pharmingen, BD Biosciences, Heidelberg, Germany). RANKL expression was evaluated after incubation with biotinylated Ab against mouse RANKL (clone IK22/5; Biolegend) or biotinylated rat IgG2a (isotype control; Biolegend) followed by avidin-fluorescein isothiocyanate (FITC) staining (4 *μ*g/sample, R&D Systems, Wiesbaden-Nordenstadt, Germany) [21]. The samples were analyzed with flow cytometer (BD LSR II) using BD FACSDiva v6.1.2 Software (Becton Dickinson GmbH, San Jose, CA, USA).

### 2.6. Purification and Activation of Blood Neutrophils

Neutrophils were purified from heparinized blood as described previously [25]. Cell population consists of >95% viable cells and of 89-90% positive cells for Ly6G and CD11b. Neutrophils were resuspended at concentration of 1 × 10^6^/mL in sterile complete RPMI-1640 medium (Biowhittaker; Lonza, Basel, Switzerland) containing 10% FCS, 2 mM L-glutamine, 100 U/mL penicillin, 100 *μ*g/mL streptomycin (all from Sigma-Aldrich), and granulocyte-macrophage colony-stimulating factor (GM-CSF; 50 ng/mL; PeproTech EC, London, UK). The cells were stimulated with zymosan (20 *μ*g/mL; Sigma-Aldrich) in the absence or presence of IL-17 (40 ng/mL; Abcam). After 24 hours, 37°C, cells were harvested, washed, and analyzed for RANKL expression and intracellular cytokine production. OPG concentrations in culture supernatants were also measured.

### 2.7. Intracellular Flow Cytometry

Neutrophils or synovial cells (1 × 10^6^/mL) were stimulated with phorbol myristate acetate (PMA; 10 ng/mL; Sigma-Aldrich) and ionomycin (2 *μ*M; Sigma-Aldrich) in the presence of brefeldin (GolgiStop, BD Pharmingen) for 4 hours. Cells were harvested, washed, stained with antibody against Ly6G, then fixed, and permeabilized (BD Cytofix/Cytoperm kit, BD Biosciences). After incubation with Abs against IL-17 (clone TC11-18H10), IFN-*γ* (clone XMG1.2), and appropriate isotype controls (all from BD Pharmingen), cells were subjected to flow cytometry analysis.

### 2.8. Immunoblotting

Blood neutrophils (1 × 10^6^/mL) were stimulated with zymosan (20 *μ*g/mL) and GM-CSF (50 ng/mL) for 10 min, 37°C in the absence or presence of IL-17 (40 ng/mL). Cells were washed with ice-cold PBS and lysed for 15 min on ice with buffer containing 10 mM HEPES (pH 7.9), 1.5 mM MgCl_2_, 10 mM KCl, 0.5 mM DTT, 0.5% NP-40, 0.5 mM phenylmethanesulfonylfluoride (PMSF), 1 mM Na_3_VO_4_, 5 mM NaF, and 1 *μ*g/mL protein kinase inhibitor cocktail (all from Sigma-Aldrich). Cell lysates were centrifuged at 13 000 g, 4°C. Supernatants were discarded, and cell pellets were resuspended and incubated for 1 h on ice in buffer containing 20 mM HEPES (pH 7.9), 1.5 mM MgCl_2_, 420 mM NaCl, 0.2 mM EDTA, 25% v/v glycerol, 0.5 mM PMSF, and 1 *μ*g/mL protein kinase inhibitor cocktail and centrifuged at 13 000 g, 4°C. Cell lysates (20 *μ*g/line) were separated by 10% SDS/PAGE gel electrophoresis and transferred onto nitrocellulose membrane (Thermo Scientific, Rockford, IL, USA). After blocking with 5% BSA/PBS buffer, the membranes were probed overnight with Abs against methyl histone H3 (mono methyl K9, 1 : 500 diluted, Abcam) or lamin, nuclear lamina protein (clone C-20, Santa-Cruz Biotech, Heidelberg, Germany). After washing, immunoblots were incubated with peroxidase-conjugated anti-rabbit IgG (Fab_2_) antibody (1 : 1000 diluted; Abcam) and then developed using a chemiluminescent substrate kit (Sigma-Aldrich). Protein band density was analyzed by ImageJ 1.42 software (Research Services Branch, NIH, Bethesda, MD, USA). In each sample H3K9 lines were normalized to that of lamin and presented in units.

### 2.9. Statistical Analysis

Statistical analysis was accomplished by InStat3.0 and GraphicPad Prism 5.0 software (GraphPad Software, La Jolla, CA, USA). Data were expressed as mean ± SEM. Kruskal-Wallis and Mann-Whitney* U*-tests were performed to compare the histological scores and the percentages of cartilage erosion between groups and to calculate statistical significance of the differences. For other data, the differences in the mean values between groups were analyzed with the two-tailed Student's *t*-test. Differences were considered significant when *P* < 0.05.

## 3. Results

### 3.1. Neutrophils Depletion in Arthritic Mice Decreases IL-17 and OPG Amounts in Synovial Fluid and Serum

**Figure 1:**
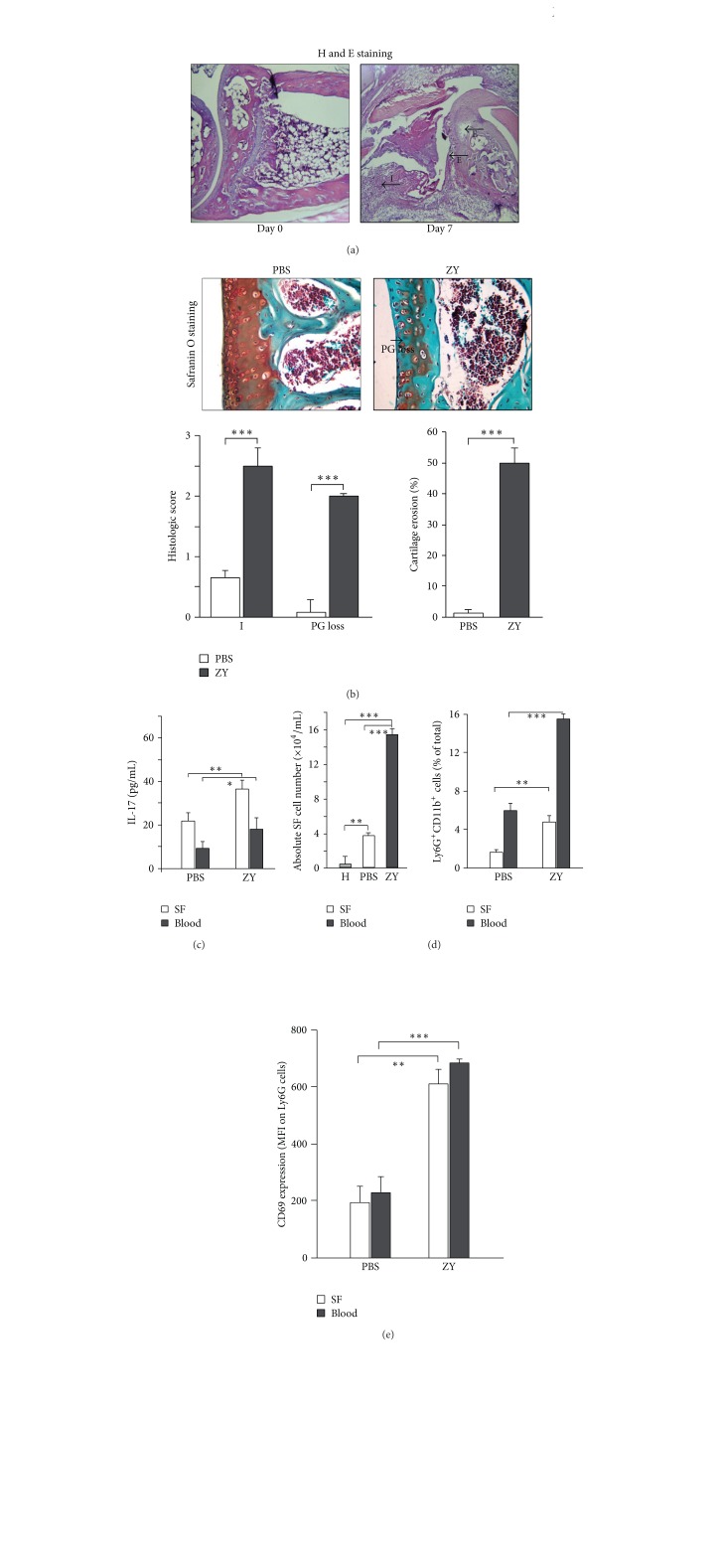
Arthritis induced by intra-articular TLR2 ligand (zymosan; ZY) injection into BALB/c mice. (a) Representative photomicrographs of hematoxylin and eosin (H&E) stained ankle sections show cell infiltration (arrow, I) and cartilage erosion (arrows, E) at day 7 of zymosan injection (200 *μ*g/10 *μ*L per ankle) (magnification 40x). (b) Photomicrographs of Safranin O stained sections (magnification 100x) and scores for cell infiltration (I), proteoglycan loss (PG loss), and cartilage erosion indicates severe joint injury in zymosan-injected mice (ZY) in comparison to PBS-injected group (PBS). Values are the mean ± SEM (10 sections/mouse; *n* = 10 mice/group; 5 experiments). ***P < 0.001, Kruskal-Wallis and Mann-Whitney* U*-test. (c) Increased IL-17 amounts in SF and serum of mice with arthritis (day 7). Bars show the mean ± SEM (*n* = 5 mice/group; 3 experiments). *P < 0.05; **P < 0.01, Student's *t*-test. (d) Cells accumulate in SF and frequencies of Ly6G^+^CD11b^+^ cells increase in SF and blood at day 7 of arthritis. Values are the mean ± SEM (*n* = 10 mice/group; 5 experiments). **P < 0.01; ***P < 0.001 versus healthy (H) or versus PBS-injected groups (PBS), Student's *t*-test. (e) Blood and SF Ly6G^+^ cells upregulate surface CD69 at day 7 of arthritis. Values are the mean ± SEM (*n* = 5 mice/group; 3 experiments). **P < 0.01; ***P < 0.001, Student's *t*-test. MFI: mean fluorescence intensity.

TLR2-driven arthritis was induced by i.a. injection of zymosan into BALB/c mice. Histological evaluation of H&E and Safranin O stained joint sections showed cell infiltration, cartilage erosion, and proteoglycan loss at day 7 of arthritis induction (Figures 1(a) and 1(b)). The amount of IL-17 raised in SF and serum of arthritic mice (Figure 1(c)). Cells accumulated in SF (Figure 1(d)). We observed increased frequencies of Ly6G^+^CD11b^+^ neutrophils in SF and blood (Figure 1(d)). CD69, characteristic for active and primed cell state, was upregulated on SF and blood Ly6G^+^ cells from zymosan-injected mice (Figure 1(e)).

**Figure 2:**
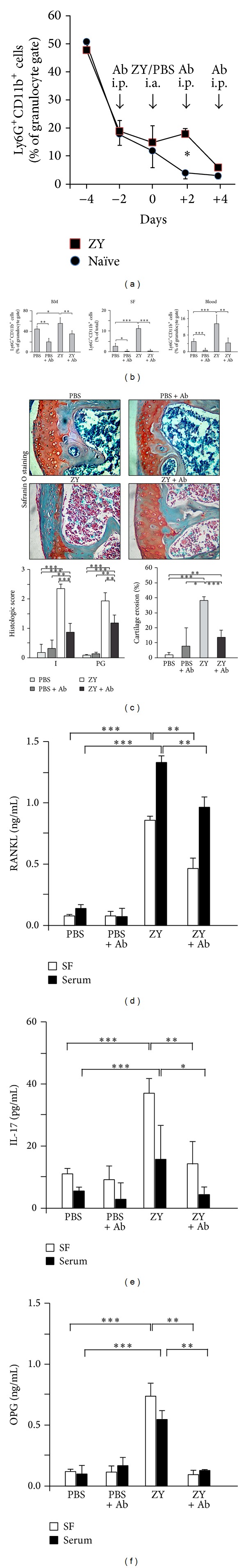
Depletion of Ly6G^+^CD11b^+^ cells with monoclonal 1A8 antibody (Ab). (a) SCID mice were injected intraperitoneally (i.p) with 1A8 Ab (100 *μ*g/mouse) at days −2, +2, and +4 (arrows showing the injections). The mice received intra-articular (i.a.) knee injection of PBS (10 *μ*L; naive) or zymosan (200 *μ*g/10 *μ*L; arrow ZY) at day 0. Flow cytometry analysis indicates the loss of Ly6G^+^CD11b^+^ cells in BM after Ab treatments. Values are the mean ± SEM (*n* = 7 mice/group), Student's *t*-test. (b) At day 7 of TLR2 ligand injection (or 3 days after the last 1A8 Ab administration) Ly6G^+^CD11b^+^ cells partially recover in BM but are completely lost in blood and SF of 1A8 Ab-treated mice. Bars indicate the mean ± SEM (*n* = 7 mice/group). *P < 0.05; **P < 0.01; ***P < 0.001, Student's *t*-test. (c) The administration of 1A8 Ab attenuates joint damages as shown on the representative photomicrographs (magnification 100x) and by histological scores for cell infiltration (I), proteoglycan (PG) loss, and percentages of cartilage erosion. Values are the mean ± SEM (*n* = 7 mice/group). **P < 0.01; ***P < 0.001, Kruskal-Wallis test and Mann-Whitney* U*-test. Ly6G^+^ cell depletion decreases the amounts of RANKL (d), IL-17 (e), and OPG (f) in serum and SF of ZY mice. Values in (d), (e), and (f) are the mean ± SEM (*n* = 7 animals/group). *P < 0.05; **P < 0.01; ***P < 0.001, Student's *t*-test.

Next we designed an experiment for neutrophil depletion by specific 1A8 Ab. SCID mice were used in these settings because they lack mature T and B cells but have intact innate immunity. The mice were treated with monoclonal 1A8 Ab at days −2, +2, and +4 of zymosan injection (Figure 2(a)).

This schedule was chosen because TLR2 ligand induced granulopoiesis even after initial 1A8 Ab administration (Figure 2(a), day +2) and the loss of Ly6G^+^CD11b^+^ cells in BM granulocyte subset (Figure 2(a), day −2). Thus cell depletion was maintained by additional Ab treatments at days +2 and +4 (Figure 2(a)). At day 7 of arthritis and 3 days after the last administration of 1A8 Ab, the neutrophils partially recovered in BM but were still absent from the circulation and SF (Figure 2(b)). Histological evaluation of the joint sections at day 7 showed a considerable decrease in cell infiltration, PG loss, and cartilage erosion in 1A8 Ab-treated ZY group (Figure 2(c)). Disease improvement was associated with diminished amounts of RANKL in serum and SF (Figure 2(d)). The administration of 1A8 Ab completely inhibited TLR2 ligand-induced production of IL-17 (Figure 2(e)) and OPG (Figure 2(f)) suggesting that Ly6G^+^CD11b^+^ neutrophils might be the source of both mediators in circulation and SF. This notion, however, posed more questions about the ability of Ly6G^+^CD11b^+^ neutrophils to produce IL-17 and OPG in response to TLR2 stimulation.

### 3.2. Altered IL-17 and IFN-*γ* Production of Blood Neutrophils from Arthritic Mice in Response to TLR2 Stimulation In Vitro

**Figure 3:**
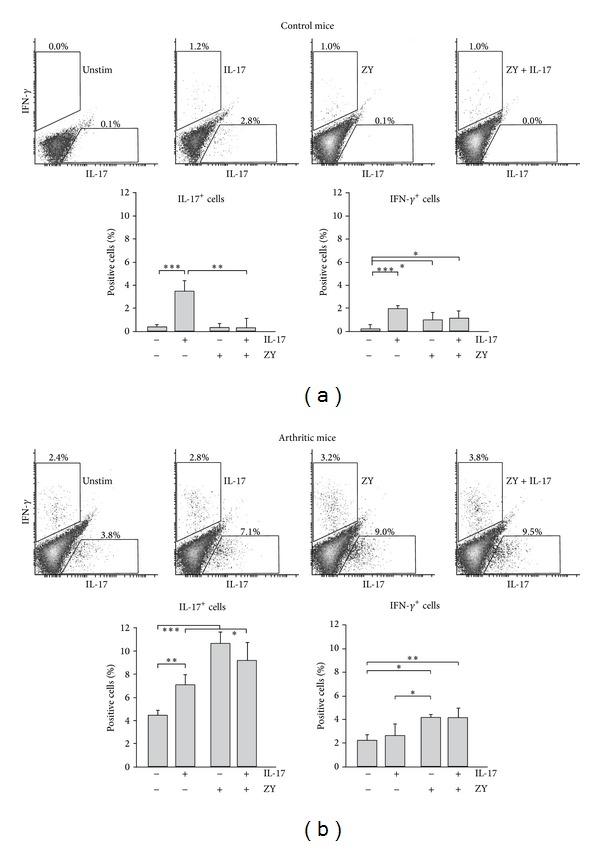
Ly6G^+^CD11b^+^ cells from arthritic mice produced IL-17 and IFN-*γ*. Purified blood neutrophils were cultured (1 × 10^6^/mL) in medium with GM-CSF (50 ng/mL) and stimulated with zymosan (20 *μ*g/mL) or/and IL-17 (40 ng/mL) for 24 hours. Intracellular IL-17 and IFN-*γ* production was evaluated by flow cytometry. (a) Ly6G^+^CD11b^+^ cells were IFN-*γ* ^−^/IL-17^−^ as shown on dot-plot histograms and graphs. IL-17 induces autocrine IL-17 protein expression and IFN-*γ* synthesis in control cells. (b) Dot-plot histograms and graphs show spontaneous IL-17 and IFN-*γ* production by Ly6G^+^CD11b^+^ cells from arthritic group. Zymosan provides stronger signals for IL-17 synthesis than exogenous IL-17 and amplifies the generation of IL-17^+^ cells. Bars on graphs (a) and (b) represent the mean ± SEM (*n* = 5 animals/group; 3 experiments). *P < 0.05; **P < 0.01; ***P < 0.001, Student's *t*-test.

Neutrophils in circulation of arthritic mice can be a source of proinflammatory cytokines as they were activated and/or primed and expressed the early activation marker CD69 (see Figure 1(e)). We purified neutrophils from blood of control (PBS-injected) and arthritic BALB/c mice. The cells were cultured in the presence of GM-CSF, a factor sustaining cell survival. Ly6G^+^CD11b^+^ cells were stimulated in vitro with TLR2 ligand and/or IL-17 for 24 hours. The intracellular production of IL-17 and IFN-*γ* was evaluated by flow cytometry (Figures 3(a) and 3(b)).

Ly6G^+^CD11b^+^ cells from nonarthritic mice were IFN-*γ* ^−^/IL-17^−^, IL-17 induced autocrine IL-17 protein expression and IFN-*γ* production in control neutrophils (representative dot-plots and graphs, Figure 3(a)). Zymosan was able to prime the control neutrophils for IFN-*γ* synthesis but it failed to initiate IL-17 production even in the presence of exogenous IL-17 (Figure 3(a)).

Neutrophils from arthritic mice produced IL-17 and IFN-*γ* spontaneously unlike the cells from the control group (Figure 3(b)). We detected around 4% IL-17^+^ cells and near 2% IFN-*γ* ^+^ cells (dot-plot histograms and graphs, Figure 3(b)). IL-17^+^ but not IFN-*γ* ^+^ neutrophils were influenced by exogenous IL-17 in the cultures (Figure 3(b)). Zymosan provided stronger signal for IL-17 synthesis and amplified the generation of IL-17^+^ cells more efficiently (Figure 3(b)). However, TLR2 enhanced the frequencies of IFN-*γ* ^+^ neutrophils, but it failed to potentiate further IFN-*γ* synthesis in the presence of IL-17. Together our data demonstrated (i) that blood neutrophils from arthritic mice have an increased potential to produce IL-17 and IFN-*γ* in comparison with control cells and (ii) that TLR2 is necessary to be stimulus (signal) for enhanced generation of IL-17^+^ and IFN-*γ* ^+^ neutrophils in cultures from arthritic group.

**Figure 4:**
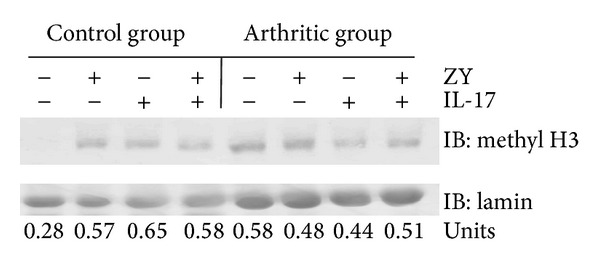
Methylated H3K9 (methyl H3) in nuclear extracts from neutrophils. Ly6G^+^CD11b^+^ cells were purified and stimulated with (20 *μ*g/mL) or/and IL-17 (40 ng/mL) for 10 min. Nuclear extracts were obtained as described in Section 2. Methylated H3K9 in nuclear extracts from neutrophils was detected by immunoblot (IB). Lamin in nuclear extracts was used as a control for protein content. Densitometry analyses of IBs were performed by ImageJ 1.42 software (NIH, Bethesda, MD, USA). The density of methyl H3K9 lines was normalized to lamin expression in each sample and was presented in units.

Neutrophil function can be regulated by epigenetic mechanisms involving methylation and acetylation of histones. We evaluated by immunoblot the levels of monomethylated H3K9 in blood neutrophils activated with zymosan and/or IL-17. Methylated H3K9 was undetectable in the nuclear extracts of nonstimulated Ly6G^+^CD11b^+^ cells from control mice and present in those from arthritic group (Figure 4).

IL-17 elevated the levels of the methylated protein in neutrophils from control but not from arthritic mice (Figure 4). These data suggest that TRL2 and IL-17 pathways might have an important impact in the epigenetic control of neutrophil functions and cytokine production. In the context of our studies they could crosstalk and compete to regulate neutrophils activities in health and disease.

### 3.3. TLR2 Ligand and IL-17 Regulate RANKL Expression and OPG Secretion by Neutrophils

**Figure 5:**
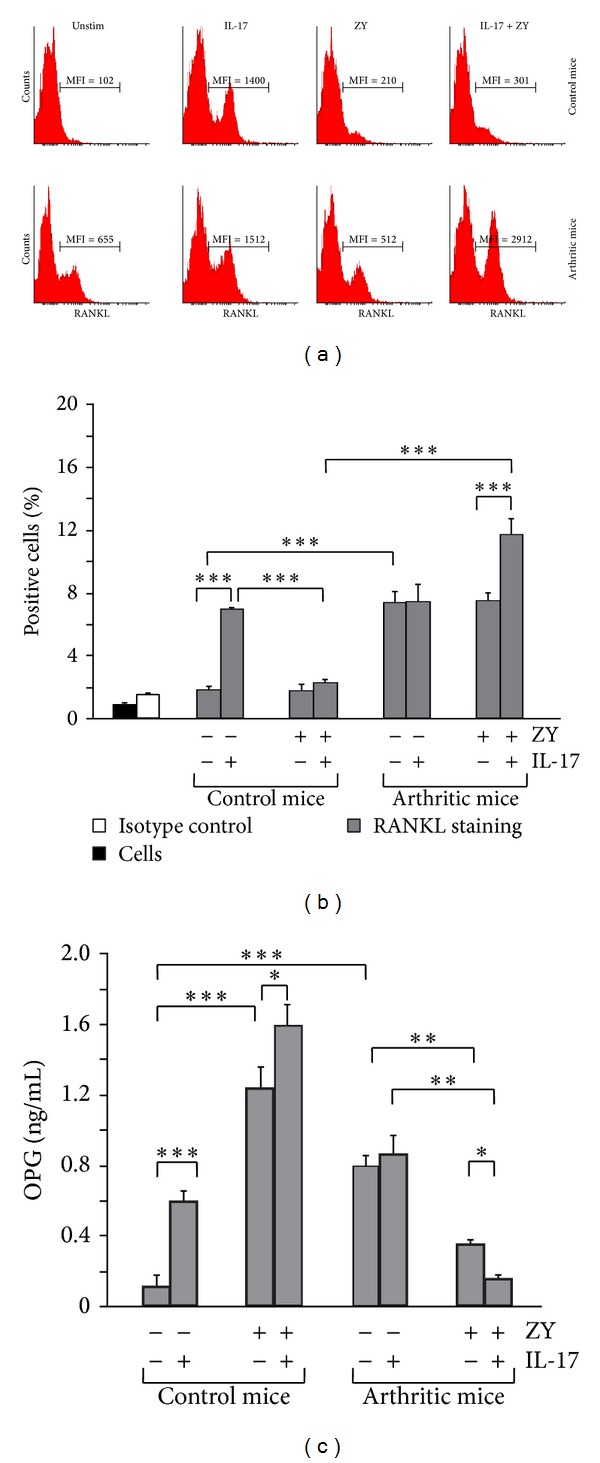
RANKL expression and OPG secretion by neutrophils were regulated by TLR2 ligand and IL-17. Purified neutrophils were stimulated as in Figure 3. (a) Representative histograms show RANKL expression on blood neutrophils from nonarthritic and arthritic mice. MFI: mean fluorescence intensity. (b) Graphs indicate that IL-17 increases the frequencies of RANKL^+^ neutrophils in the control group. More RANKL^+^ cells are found after stimulation with TLR2 and IL-17 in arthritic group. (c) IL-17 and zymosan induce OPG secretion by control neutrophils and inhibit OPG production by cells from arthritic mice. Values in (b) and (c) are the mean ± SEM (*n* = 5 animals/group; 3 experiments). *P < 0.05; **P < 0.01; ***P < 0.001, Student's *t*-test.

At day 7 of arthritis induction, the level of IL-17 in serum was elevated (Figure 1(c)) and the frequencies of IL-17^+^ neutrophils were enhanced in the circulation (Figure 3(b)). In order to study how this altered IL-17 production influences the destructive potential of neutrophils, we analyzed the expression of RANKL, a molecule directly involved in bone erosion and resorption. Blood Ly6G^+^CD11b^+^ cells from nonarthritic mice did not express RANKL (histograms, Figure 5(a)). Exogenous IL-17 induced RANKL expression (histograms, Figure 5(a)) and increased the frequencies of RANKL^+^ neutrophils in the control group (graph, Figure 5(b)). However, TLR2 ligand failed to trigger RANKL expression on neutrophils from control mice even in the presence of IL-17 (histograms, Figure 5(a) and graph, Figure 5(b)).

Blood Ly6G^+^CD11b^+^ cells from arthritic group expressed RANKL unlike neutrophils from nonarthritic mice. Exogenous IL-17 increased the intensity of RANKL staining but not the frequencies of RANKL^+^ neutrophils (Figures 5(a) and 5(b)). Zymosan failed to change RANKL expression but yet in combination with IL-17 enhanced substantially RANKL staining intensity and the frequencies of RANKL^+^ cells (Figures 5(a) and 5(b)).

In the same experimental setting we evaluated the amount of secreted OPG in culture supernatants (Figure 5(c)). Blood neutrophils from nonarthritic mice produced OPG upon IL-17 and TLR2 ligand stimulation alone or in combination (Figure 5(c)). Ly6G^+^CD11b^+^ cells from arthritic mice secreted more OPG in cell cultures than the control cells (Figure 5(c)). By contrast to controls exogenous IL-17 failed to elevate OPG production by neutrophils from arthritic group. The engagement of TLR2 ligand diminished OPG secretion in cultures of Ly6G^+^CD11b^+^ cells from arthritic mice. This effect was amplified by IL-17 (Figure 5(c)), simultaneously to the upregulated RANKL expression on Ly6G^+^CD11b^+^ cells (Figures 5(a) and 5(b)).

### 3.4. RANKL Expression, IL-17, and IFN-*γ* Production of Ly6G^+^ Cells in SF

**Figure 6:**
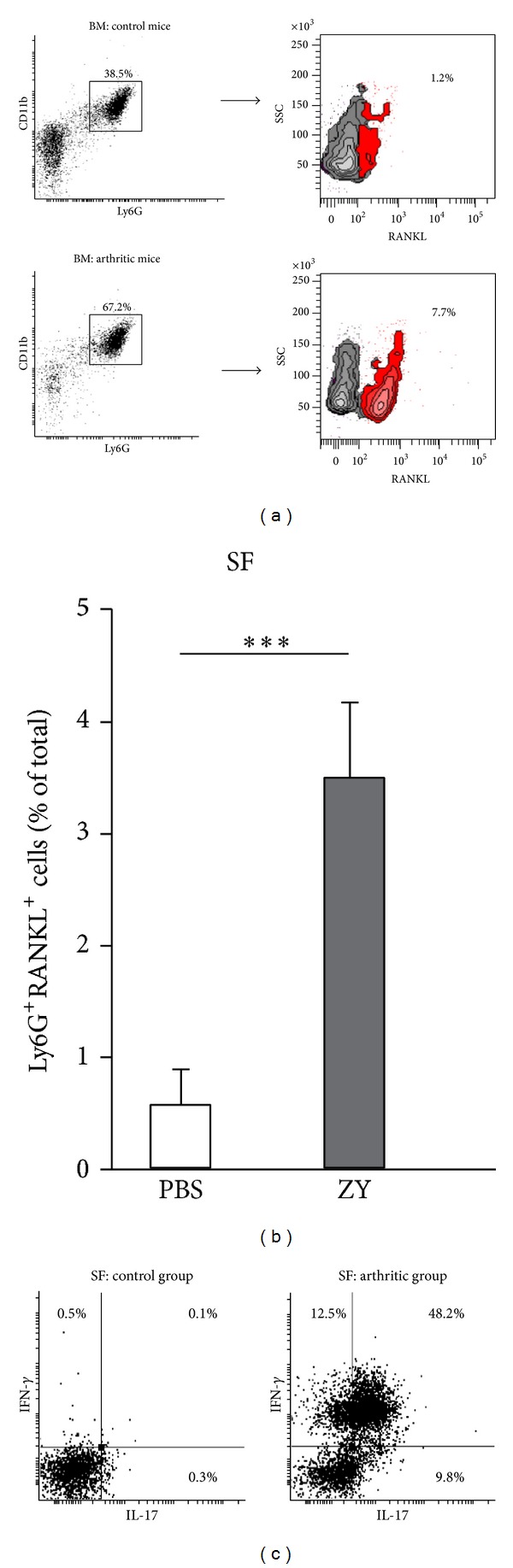
RANKL expression, IL-17, and IFN-*γ* production of Ly6G^+^ cells in BM or SF. (a) Dot-plot histograms show increased frequencies of Ly6G^+^CD11b^+^ cells in BM of mice with arthritis than in control group. The density plots indicate a higher distribution of RANKL^+^ cells in Ly6G^+^CD11b^+^ population from arthritic group (SSC: side scatter). (b) RANKL-bearing Ly6G^+^ cells accumulated in SF of mice with arthritis. Bars represent the mean ± SEM (*n* = 5 animals/group; 3 experiments). ***P < 0.001, Student's *t*-test. (c) Synovial cells from 5 animals per group were pooled and intracellular cytokine production was performed on gated Ly6G^+^ cells. Representative dot-plot histograms show IFN-*γ* and IL-17 production in Ly6G^+^ cells from SF of mice with arthritis (day 7) (*n* = 5 animals/group; representative from 3 experiments).

In vivo RANKL^+^ neutrophils in blood and SF of arthritic mice can originate from mature neutrophils in BM. We found more RANKL^+^ cells in BM Ly6G^+^CD11b^+^ population from arthritic mice in comparison with control (Figure 6(a)). RANKL-bearing Ly6G^+^ cells accumulated in SF of mice with arthritis (Figure 6(b)).

The cytokine production of synovial Ly6G^+^ cells was also studied. The cell number yield from each mouse was low to run intracellular flow cytometry. Thus, we pooled the SF cells from five mice per group and stained them for Ly6G, IFN-*γ*, and IL-17. Flow cytometry analyses were performed on gated Ly6G^+^ population. Synovial Ly6G^+^ cells from arthritic mice expressed IFN-*γ* and IL-17 unlike the cells from control mice (Figure 6(c)). It appeared that most of the cells were IFN-*γ* ^+^IL-17^+^ (Figure 6(c)).

## 4. Discussion

The role of neutrophils in joint diseases has been mainly associated with the secretion of proteolytic enzymes and reactive oxygen radicals. Our study showed that the bone destructive potential of neutrophils in arthritis was sustained by increased IL-17 production and RANKL expression and inhibited OPG secretion. In vitro exogenous IL-17 enhanced the functional activity (IL-17 and IFN-*γ* production and RANKL expression) of blood neutrophils from both control and arthritic mice. The effects of IL-17 were amplified by TLR2 ligation on Ly6G^+^CD11b^+^ cells from arthritic mice only. Therefore, we conclude that targeting TLR2 signaling on neutrophils may limit bone resorption and joint damage in arthritis.

Neutrophils arise from granulocyte precursors in BM. Mature BM Ly6G^+^CD11b^+^ cells maintain the replenishment pools in blood and spleen [25]. Various proinflammatory factors and mediators trigger granulopoiesis and enhance the mobilization of mature Ly6G cells from BM [2]. Among them is TLR2 which regulates neutrophil release via transcriptional upregulation of G-protein-coupled receptor kinase-2 and chemokine receptor CXCR2 downregulation [26]. In our model the stimulatory effects of TLR2 on granulopoiesis and neutrophils trafficking was confirmed by increased frequencies of Ly6G^+^CD11b^+^ cells in BM, blood, and SF and by CD69 expression on circulating neutrophils. In vitro neutrophils upregulate CD69 in response to various stimuli that induce cell activation or priming like TLRs, TNF-*α*, GM-CSF, IFN-*γ*, or IFN-*α* [27].

The depletion of neutrophils by specific antibody shows a crucial role of neutrophils for disease progression in a model of collagen-antibody induced arthritis [28]. Two clones of Abs are available for neutrophil elimination, clones RB6-8C5, and 1A8. While 1A8 Ab recognizes Ly6G, RB6-8C5 binds to two Ly6 isoforms, Ly6G, and Ly6C. Besides on neutrophils, Ly6C is found on dendritic cells, subpopulations of lymphocytes, and monocytes [28]. We used more a specific 1A8 clone to target the population of mature neutrophils. Ly6G^+^CD11b^+^ cells disappeared from blood and SFs of 1A8 Ab-treated mice. The administration of 1A8 Ab reduced the degree of joint damages as shown by decreased scores for cell infiltration, cartilage erosion, and PG loss and diminished amount of bone-erosion accelerating marker RANKL in SF and serum. 1A8 Ab completely abrogated OPG and IL-17 production indicating that Ly6G^+^CD11b^+^ cells were the source of both factors in serum and SF. We think that neutrophils may provide a certain level of IL-17 that later on during the development of arthritis can be amplified by mast cells, monocytes, or T cells secreting also IL-17 [29, 30]. In RA patients as well as in our study IL-17 appears at initial stage of disease and before the disease onset [9–11]. Moreover, the level of IL-17 in SF can predict the progression of joint damage in RA patients [12].

Three recent studies have shown that neutrophils produced IL-17 under infectious and allergic conditions [31–33]. Intracellular IL-17 production was detected in synovial and blood Ly6G^+^CD11b^+^ cells from arthritic BALB/c mice. IL-17 protein synthesis is regulated by IL-17 gene as well as of genes for key transcription factors. Transcription is fine-tuned by epigenetic histone modifications such as methylation and acetylation. We observed the expression of monomethylated H3K9 in neutrophils from arthritic mice but not in control cells. The methylation of H3K9 induces gene silencing and is associated with human and mouse granulocytes differentiation and abnormalities in myeloid leukemia [34, 35]. In our study IL-17 triggered its own production and H3K9 methylation in nonarthritic neutrophils proposing that certain genes are silenced in order to acquire IL-17 expression. Epigenetic modification of IL-17 gene expression is mainly studied in T cells. Considerable amounts of IL-17 can be maintained in T cells by increasing histone H3 acetylation and methylation at the IL-17 gene promoter [36]. Our data are too preliminary, but they outline the interest to study IL-17 transcription and epigenetic modification of IL-17-dependent gene expression in neutrophils and granulocytes and in neutrophils in particular.

Ly6G^+^CD11b^+^ cells from nonarthritic and arthritic mice were sensitive to IL-17 stimulation, but they responded differently to simultaneous IL-17R and TLR2 ligation in vitro. While zymosan blocked the effects of exogenous IL-17 on neutrophils from nonarthritic mice, it amplified IL-17 production in Ly6G^+^CD11b^+^ cells from arthritic group. TLR2 and IL-17 signaling pathways can interfere at various levels. In particular, a conserved motif in the cytoplasmic domain of receptor for IL-17 with homology to the TIR domain has been identified [37]. TIR domain has a specific docking site for adaptor protein MyD88 that allows the involvement of TLR2 signaling pathway. Common transduction proteins can sustain the assembly of IL-17R and can regulate the strength of receptor expression [38]. At transcriptional level both pathways can interfere through NF-*κ*B activation and pathway (reviewed by [39]). We speculated that TLR2 and IL-17R pathways directly compete for intracellular kinases, adaptor proteins, or transcription factors. In neutrophils from nonarthritic mice activated molecules or factors were limited and less available for both signaling. By contrast Ly6G^+^CD11b^+^ cells from arthritic group were primed or activated (as shown by the expression of CD69) and in turn they have in disposal high numbers of common transduction molecules and transcription factors. Thus both pathways via their crosstalk can provide a mechanism for regulation of neutrophils activities in health and disease.

IL-17 increases the recruitment of neutrophils at the site of inflammation and influences the production of various proinflammatory mediators [11]. We observed that IL-17 initiated IFN-*γ* production in Ly6G^+^CD11b^+^ cells from nonarthritic mice but failed to increase the frequencies of IFN-*γ* ^+^ cells in arthritic group. IL-17 can reduce degradation of mRNA for certain cytokines and can enhance cell responsiveness to second stimuli [40]. Indeed IL-17-producing neutrophils act proximally and are required for IFN-*γ* production [33]. In vivo TLRs, various proinflammatory cytokines, cell environment, and disease stage can elicit the action of IL-17 on neutrophils. In arthritic synovium IL-17 activates fibroblasts and synoviocytes to produce IL-6, IL-8, TNF-*α*, and GM-CSF and to express TLR2 favoring cytokine production, activation, and survival of neutrophils [41, 42]. In such environment most of Ly6G^+^ cells in SF were double positive for IFN-*γ* and IL-17. The specific factors and cell populations in blood created the conditions that likely generated single IFN-*γ* ^+^ or IL-17^+^ neutrophils. In cell cultures from arthritic group TLR2 restimulation sustained this phenotype and enhanced the generation of single IFN-*γ* ^+^ and IL-17^+^ cells.

Bone destructive processes such as bone erosion and bone resorption depend on the activation of osteoclasts. These cells are sensitive to the action of IFN-*γ* and IL-17, but the RANKL/OPG system is crucial for their differentiation and maturation. Various studies show a correlation between severity of bone diseases and RANKL/OPG ratio in serum and SF (reviewed by [43]). OPG is a soluble protein from the TNF receptor superfamily and it inhibits osteoclast differentiation and activity. We assume that neutrophils at the initial stage of disease secreted OPG because the molecule was absent in serum and SF after cell depletion by 1A8 Ab. Experiments in vitro confirmed that OPG was released by blood neutrophils from nonarthritic mice upon TLR2 stimulation or spontaneously by neutrophils from TLR2 ligand-injected group. Ly6G^+^CD11b^+^ cells from arthritic mice failed to produce OPG after zymosan restimulation. This altered responsiveness to repeated stimulation might maintain low OPG levels during the development of arthritis that in turn can sustain osteoclastogenesis and osteoclast activation. We think that such mechanism may dominate at late stages of disease when more neutrophils are accumulated in SF.

OPG decoy receptor, RANKL, exists in two isoforms, a soluble protein, and a membrane bound protein. The latter is sensitive to the cleavage by proteases. Thus, the neutrophils producing proteases may affect the amount of soluble RANKL in biological fluids. Indeed we found that after neutrophil depletion the concentrations of RANKL decreased in serum and SF of zymosan-injected mice.

The membrane bound RANKL is expressed by BM cells, precursors of osteoclasts, and stromal cells [43]. In vivo TLR2 ligand induced RANKL expression on Ly6G^+^CD11b^+^ cells in BM. Despite that RANKL^+^ pool of Ly6G^+^ cells was small, it probably maintained the frequencies of RANKL^+^ neutrophils in circulation and even contributed to the accumulation of RANKL-bearing cells in SF at early stage of disease (day 7). The study describing RANKL expression on blood neutrophils from RA patients supported our data [19]. The same authors indicated the role of the environmental factors since neutrophils from healthy donors upregulated RANKL after incubation with SF from RA patients [19]. We found that exogenous IL-17 induced RANKL expression on RANKL-negative blood neutrophils (control) and increased the density of surface RANKL on RANKL-bearing Ly6G^+^CD11b^+^ cells (arthritic group). TLR2 engagement potentiated the IL-17-mediated RANKL expression only on neutrophils from arthritic mice and inhibited OPG secretion in cell cultures at the same time. Thus we built the hypothesis that TLR2 signaling sustained the bone destructive potential of neutrophils by an interference with IL-17-dependent RANKL/OPG system.

In summary, our study showed that (i) exogenous IL-17 induced autocrine IL-17 production, IFN-*γ* synthesis, and RANKL expression on blood neutrophils from nonarthritic mice and these effects were inhibited upon simultaneous TLR2 stimulation; (ii) Ly6G^+^CD11b^+^ cells from arthritic group produced IL-17, IFN-*γ*, and OPG spontaneously and expressed RANKL; (iii) TLR2 increased IL-17-mediated RANKL expression and inhibited OPG secretion by neutrophils from arthritic mice. Together these data suggest that TLR2 signaling might be a good target to limit the bone destructive potential of neutrophils in joint diseases.
